# Retrospective PCR-based species identification of *Leishmania* in two patients with visceral leishmaniasis in Serbia

**DOI:** 10.1099/jmmcr.0.005063

**Published:** 2016-10-20

**Authors:** Zorica Dakić, Henrik Vedel Nielsen, Milorad Pavlović, Jasmina Poluga, Goran Stevanović, Lidija Lavadinović, Branko Milošević, Mijomir Pelemiš, Aleksandar Urošević, Snežana Jovanović, Christen Rune Stensvold

**Affiliations:** ^1^​ Parasitological Laboratory, Department of Microbiology, Clinical Centre of Serbia, Bulevar Oslobodjenja 16, 11000 Belgrade, Serbia; ^2^​ Laboratory of Parasitology, Department of Microbiology and Infection Control, Division of Diagnostics and Infection Control, Statens Serum Institute, 5 Artillerivej, DK–2300 Copenhagen, Denmark; ^3^​ Medical Faculty, University of Belgrade, Dr Subotića 8, 11000 Belgrade, Serbia; ^4^​ Clinic for Infectious and Tropical Diseases, Clinical Centre of Serbia, Bulevar Oslobodjenja 16, 11000 Belgrade, Serbia

**Keywords:** visceral leishmaniasis, PKDL, amastigotes, molecular diagnostic, sequencing, *Leishmania infantum*

## Abstract

**Introduction:**

Retrospective molecular identification of *Leishmania *parasites in two patients with visceral leishmaniasis (VL) previously treated in Serbia was carried out. DNA was isolated from unstained bone marrow smears (BMSs) kept for 11 and 8 years. Genus-specific real-time PCR was combined with conventional PCR and sequencing for detection and species identification.

**Case presentation:**

In 2003, a 40-year-old Serbian male was admitted to the Clinical Centre of Serbia (CCS) with fever, sweating, fatigue and splenomegaly, which developed over a period of 7 weeks. He had frequently travelled around Europe. VL was confirmed by microscopy of Giemsa-stained BMS. Treatment by pentavalent antimonials was successfully completed. Two years later, the patient developed post-kala-azar dermal leishmaniasis. Treatment resulted in symptom resolution. Later on, *Leishmania infantum* was identified as the causative agent of the VL by sequencing of the ITS (internal transcribed spacer) region; mixed *Leishmania* spp. infection could not be excluded. In 2006, a 33-year-old female from Vojvodina, Serbia, with pre-existing diabetes mellitus and chronic meningoencephalitis and a history of frequent visits to the Montenegrin seacoast, was admitted to the CCS with fever, pancytopenia and moderate hepatosplenomegaly. A stained BMS revealed abundant *Leishmania* amastigotes. Indirect haemagglutination analysis was positive with a titre of 1 : 2048, and a rapid dipstick rK39 test was also positive. Treatment by liposomal amphotericin B was successful; however, shortly after, the patient developed neural infection and pneumonia and died. The causative agent was identified as *L. infantum*.

**Conclusion:**

Molecular diagnosis of VL and species delineation using DNA from unstained BMSs stored for several years is possible.

## Introduction

Leishmaniasis is becoming a growing health issue with increased numbers of cases reported in European areas previously not known to be endemic or being reported as a re-emerging disease in formerly endemic areas. Visceral leishmaniasis (VL) occurs sporadically in Serbia. According to epidemiological data, eight new VL cases were reported in Serbia from 2008 to 2014 (Institute of Public Health of Serbia, 2009, 2015). In central Serbia, the incidence was 0.01/100 000 in 2012 versus 0.03/100 000 in 2013. Cases of autochthonous VL have been reported in all countries neighbouring Serbia, including Montenegro, Croatia, Bosnia, Romania, Bulgaria, the FYR Macedonia, Greece and Turkey (Beatović, 2010; Durda, 2010; Gradoni, 2013; Šiško-Kraljević *et al.*, 2013).

Most patients with suspected leishmaniasis in Serbia are referred to the Clinical Centre of Serbia (CCS) for diagnosis and treatment. Our study of VL, carried out from 2001 to 2007 involving 22 VL cases, showed an inadequate sensitivity of the initial bone marrow smear (BMS) analysis (86.36 %), indicating the need for applying more sensitive methods, such as PCR (Dakić *et al.*, 2009). Over the next 8 years, from January 2008 to December 2015, nine cases (seven initial cases and two relapses) of VL were diagnosed in the Parasitological Laboratory, CCS, including one case of leishmaniasis/human immunodeficiency virus (HIV) co-infection. The majority probably contracted VL at the Montenegrin seacoast, with only one case being imported from outside the borders of the former Yugoslavia (Portugal). Reliable diagnosis of VL, including accurate species identification, is critical to ensure adequate treatment and the development of prevention measures. Unfortunately, molecular diagnostics for detection and differentiation are not yet available in Serbia.

Here, we performed retrospective molecular identification of *Leishmania* parasites in two patients previously treated in Serbia: one case of VL and one case of VL with consequent post-kala-azar dermal leishmaniasis (PKDL). DNA was isolated using a QIAamp DNA Mini kit (QIAGEN) from unstained BMSs kept for 8 and 11 years, respectively. Smears were subjected to gentle scraping using 180 µl ATL buffer solution. DNA extraction was performed according to the manufacturer’s recommendations. Extracted DNA was resuspended in 100 µl AE buffer. Positive results by genus-specific real-time PCR were confirmed by conventional PCR followed by sequencing of the internal transcribed spacer 1 (ITS1) region and blast analysis of the sequences against the National Center for Biotechnology Information nucleotide sequence database (http://www.ncbi.nlm.nih.gov/nucleotide) for species identification.

## Case report

### Case I

**Fig. 1.**
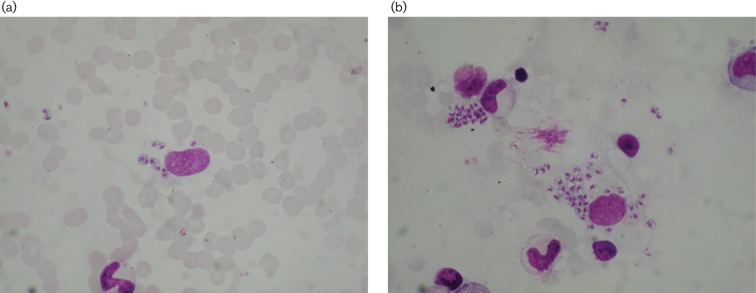
Case I: *Leishmania *species amastigotes in Giemsa-stained BMSs under oil immersion (×1000). (a) Amastigotes in the extracellular area, (b) amastigotes in the extracellular area.

In May 2003, a 40-year-old Serbian male who had been living in Sweden was admitted to the Clinic for Infectious and Tropical Diseases (CITD), CCS, in Belgrade with fever above 39 °C, sweating, fatigue and splenomegaly developing over a period of 7 weeks. Onset of a flu-like illness started on March 10 2003 with high temperature (up to 37.7 °C), sore throat, sneezing, chest pains, shortness of breath, and aches and pains in muscles and joints. He was treated at a primary health care centre during April 2003 and received repeated courses of antibiotic treatment (amoxicillin/clavulanic acid, roxithromycin, sulfamethoxazole and trimethoprim) without any improvement. On admission to the CITD, laboratory test results were as follows: erythrocyte sedimentation rate, 92 mm h^−1^; erythrocytes, 3.05×10^12^ l^−1^; haemoglobin, 122 g l^−1^; leukocytes, 2.1×10^9^ l^−1^; platelets, 129×10^9^ l^−1^. Abdominal ultrasonography showed hepatomegaly, with the right lobe having a size of 16.7 cm, and splenomegaly (length 13.7 cm). The patient had frequently travelled around Europe (Spain, Sweden, Germany), including countries in South-East Europe. VL was confirmed on May 15 2003 by examination of Giemsa-stained BMSs revealing numerous amastigotes of *Leishmania* (Fig. 1). Serology for *Leishmania* was not available at this time. Treatment by pentavalent antimonials (Glucantime) was started immediately using a dose of 20 mg kg^−1^ daily. After 3 days, the patient was requested to leave the hospital since he had no health insurance in Serbia. Treatment was successfully completed in Sweden (details of the treatment are not available). Two years later, in April 2005, during a stay in Serbia, the patient developed skin lesions. In the beginning, a diagnosis of lupus was considered. The patient was diagnosed as HIV negative. Three months later, all nodular lesions resolved. On August 01 2005, the patient visited a private dermatologist in Belgrade, Serbia, who consulted a specialist in infectious diseases who had treated the patient 2 years earlier for the VL. The specialist suggested PKDL as a diagnosis. Indirect haemagglutination analysis (IHA) for *Leishmania*-specific antibodies (Siemens) was negative, and a rapid dipstick rK39 test (DiaSys Europe) was weakly positive. A diagnosis of PKDL was established by parasitological examination for *Leishmania* amastigotes in skin-biopsy specimens performed in Serbia by a dermatologist, while the patient was treated with amphotericin B in Sweden (details of the treatment are not available). Treatment resulted in symptom resolution. Later on, in October 2014 at the Statens Serum Institute, *Leishmania infantum* was identified as the causative agent of the VL by sequencing of the ITS region; based on sequence chromatogram analysis, mixed *Leishmania* spp. infection, however, could not be excluded.

### Case II

**Fig. 2.**
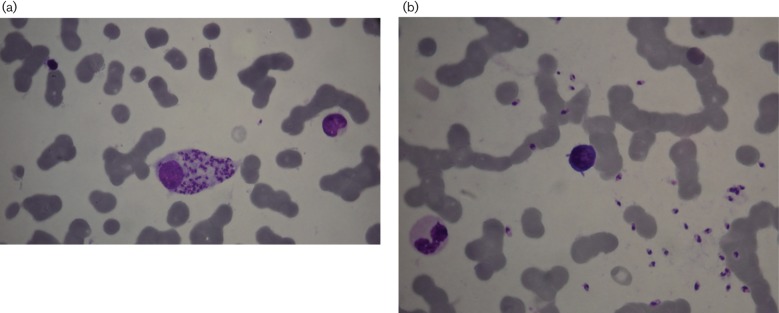
Case II: *Leishmania *species amastigotes in Giemsa-stained BMSs under oil immersion (×1000). (a) Intracellular amastigotes in macrophages, (b) amastigotes in the extracellular area.

In mid-July 2006, a 33-year-old female from Vojvodina, northern Serbia, with pre-existent congenital hydrocephalus with a ventriculoperitoneal shunt implanted during infancy, chronic meningoencephalitis, diabetes mellitus, chronic renal failure and a history of frequent visits to the Montenegrin seacoast, was admitted to the CITD with fever, pancytopenia and moderate hepatosplenomegaly. An unfamiliar feeling of weakness with chills and fever had started 2 months earlier, in mid-May 2006. The patient was admitted to the neurosurgical department at the regional hospital. First, ventriculoperitoneal shunt infection was suspected, but it was not confirmed. When pancytopenia and elevated levels of blood urea nitrogen and creatinine appeared in the patient, who previously was known as having a normal blood profile, VL was suspected. Sternal puncture was performed, and the patient was immediately transferred to the intensive care unit of the CITD. On admission, laboratory tests revealed the following values: erythrocytes, 1.85×10^12^ l^−1^; haemoglobin, 46 g l^−1^; leukocytes, 0.6×10^9^ l^−1^; platelets, 22×10^9^ l^−1^. Abdominal ultrasonography showed hepatomegaly, with the right lobe measuring 13.5 cm, and splenomegaly (length 15.0 cm). Immediately upon admission, a stained BMS revealed abundant *Leishmania* amastigotes (Fig. 2). Indirect haemagglutination testing for *Leishmania*-specific antibodies was positive with a titre of 1 : 2048, and a rapid dipstick rK39 test was also positive. Seven days of treatment with liposomal amphotericin B (AmBisome) in daily doses of 50 mg was given. Although expensive, this treatment was chosen due to high efficacy and low toxicity, good local experience and the general availability of this drug. Resolution of the symptoms was achieved during the first month after the therapy was completed. However, shortly after improvement, the patient developed neural infection and pneumonia, resulting in respiratory failure. Despite rehydration therapy and respiratory support by non-invasive ventilation, rapidly progressive deterioration resulted in respiratory and cardiocirculatory insufficiency and death 80 days after hospitalization. Molecular analysis performed in October 2014 at the Statens Serum Institute identified the causative agent as* L. infantum*.

## Discussion

At least 20 *Leishmania *species can infect humans and cause a wide spectrum of clinical diseases, including three main types: VL (the most serious form), cutaneous leishmaniasis and mucocutaneous leishmaniasis (Peacock *et al.*, 2007). VL is endemic to Mediterranean Europe, where the disease is caused by *L. infantum*.

For the last decade, stable, low-incidence rates of VL have been observed in Serbia. Insufficient research and frequent cross-travelling of residents between the former Yugoslavian republics blurs the understanding of autochthonous transmission of VL in Serbia. A few reports suggest that animal reservoirs exist. The potential epidemiological role of the golden jackal in dispersing *Leishmania *in Serbia should be taken into consideration when applying surveillance monitoring schemes. A total of 216 spleen samples from golden jackals were collected from 2010 to 2013, from 12 sites all over Serbia, with 6.9 % being positive for *Leishmania *species by quantitative PCR (qPCR) (Ćirović *et al.*, 2014). From 2009 to 2013, Savić *et al.* (2014) analysed dog blood samples for leishmaniasis, by a routine ELISA test, in the region of Vojvodina in northern Serbia. The seroprevalence was 15 % (33/220); however, 21 dogs had travelled abroad with their owners in VL-endemic areas before testing. Vaselek *et al.* (2015) performed entomological surveillance of sand flies in Vojvodina in 2013. All 55 specimens of the genus *Phlebotomus* were *Phlebotomus papatasi*, apart from a single specimen identified as *Phlebotomus tobbi*. Four specimens of *P. papatasi* were positive for *L. infantum* by nested PCR, indicating the presence of this pathogen in the host and possible transmission within Serbia. For this reason, an epidemiological survey should be performed, including investigations of potential reservoir hosts and vectors in the surroundings of people with VL, so as to clarify the epidemiology of *Leishmania* in Serbia. A recent survey performed in several endemic foci of VL in Croatia disclosed a high prevalence of asymptomatic parasite carriers (Šiško-Kraljević *et al.*, 2013).

One of the most attractive destinations for Serbian tourists is the Montenegrin seacoast; the south-coast region is considered endemic for leishmaniasis. From 1992 to 2009, 55 cases of VL were reported in Montenegro, along with one case of cutaneous leishmaniasis. From 2006 and 2009, serological surveys of clinically suspect dogs coming from VL-endemic areas identified a 58 % mean rate of infection (Beatović, 2010). The vector was *Phlebotomus neglectus* (Ivović *et al.*, 2004).

Although imperfect, parasitological examination based on demonstration of the parasites of *Leishmania* is included in the gold standard diagnostic work-up for VL. Except for the invasive procedure of sampling, microscopy is straightforward and reliable if amastigotes are plentiful and if the microscopist is experienced; however, this is often not the case. Moreover, by parasitological examination, species of *Leishmania *are indistinguishable. Together with a number of other factors (host genetic variability, specific immune response), the main factor determining the clinical presentation of infection is the species of *Leishmania *(Lipoldova & Demant, 2006). Moreover, species identification is important for treatment selection.

Generally, DNA-based methods have enabled a higher accuracy in the diagnosis of VL. Advantages of these methods include high sensitivity and specificity, and DNA-based methods can be performed on different sample materials [peripheral blood, spleen, liver, bone marrow (BM) and lymph node aspirates]. Moreover, such methods may enable distinction between relapse and reinfection, and enable the diagnosis of VL in HIV-infected patients (De Ruiter *et al.*, 2014). Using DNA-based methods, De Ruiter *et al.* (2014) showed that analysis of blood samples is as effective as analysis of (the more invasive) BM samples. In Serbia, the availability of molecular methods is limited for rare parasitic infections (including VL), which possibly has to do with cost-effectiveness issues. As of yet, the gold standard in Serbia for VL diagnosis is assessment of clinical presentation in combination with the demonstration of amastigotes in Giemsa-stained BMSs and/or positive serology. The interpretation of sequences obtained by Sanger sequencing of PCR products may be difficult in cases of mixed species infection as in case I. We believe that the use of next generation sequencing of PCR products obtained by genus-specific primers or the use of species-specific DNA-based methods, such as multiplex real-time PCR assays, could eliminate the problem of not being able to delineate species in mixed *Leishmania* infections.

There are several methods for species identification of *Leishmania*, including the use of mAbs, analysis of isoenzymes and molecular methods. The gold standard for species identification of *Leishmania* is still isoenzyme electrophoresis, while ITS1 sequencing is an alternative method (Mahmoudzadeh-Niknam *et al.*, 2011). In the first patients, mixed *Leishmania* spp. infection could not be excluded by molecular analysis. Mixed *Leishmania* spp. infection is possible as a consequence of extensive travelling. Mixing up or contamination of *Leishmania *isolates with non-*Leishmania *trypanosomatids (e.g. *Crithidia*) in some laboratories is possible too (Mahmoudzadeh-Niknam *et al.*, 2011). Weakly positive results of the dipstick rK39 test could be a consequence of low affinity or low levels of antibodies. Das *et al.* (2011) reported three cases where the rK39 strip test failed to detect two cases of PKDL and one case of VL. Haldar *et al.* (1981) showed that IHA, when used for PKDL, was positive in only one third of patients with chronic PKDL, while all fresh cases were positive, indicating antibody titre reduction during the chronic phase. This could explain the negative IHA result during PKDL in our first patient. Molecular analysis of *Leishmania* parasites from the second patient confirmed with 100 % sequence identity that *L. infantum*, probably contracted in Montenegro, was the cause of the VL.

High quality and preservation of each sample (blood, biopsies/aspirates) for *Leishmania* DNA extraction is crucial. Previous studies have shown the possibility of extracting amplifiable DNA of *Leishmania* from dried or old materials, including archival air-dried unstained BM slides or Giemsa-stained slides (Brustolini *et al.*, 2007), blood or BM aspirates spotted onto filter paper (Cortes *et al.*, 2004; Da Silva *et al.*, 2004), and even paraffin-embedded tissues (Lanús *et al.*, 2005) or formalin-fixed material (Gebhardt *et al.*, 2015). Slide smears are easily stored, do not require special storage conditions and can be kept at room temperature.

Latency in terms of establishing the diagnosis of VL was a consequence of the non-specific initial presentation of the illness (case I) or the result of pre-existing diseases mimicking VL (case II). The rare occurrence of VL explains why not all clinicians are familiar with this disease. The treatment of VL is very complex, because drug resistance has become a major obstacle to effective treatment. Traditionally, mainly antimony (Glucantime) in doses of 20 mg kg^−1 ^for 21–28 days was used as the first-line therapy for VL patients in Serbia, as it was the cheapest, low toxic and effective anti-leishmanial drug. In recent years, however, we have observed sporadic cases unresponsive to antimony therapy. In these cases, favourable outcomes have been achieved by use of liposomal amphotericin B. Considering that VL is a rare disease, anti-leishmanial drugs, excluding amphotericin B, have not yet been registered in Serbia.

PKDL is a dermal complication of VL, reported mainly in Sudan in eastern Africa and the Indian subcontinent, with incidences of up to 50–60 % and 5–10 %, respectively (Zijlstra *et al.*, 2003; Ganguly *et al.*, 2010). The pathogenesis of PKDL is mainly immunologically mediated and develops at variable intervals after/or during therapy for VL, e.g. after 0–6 months in Sudan and 2–3 years in India (Zijlstra *et al.*, 2003). Most cases of PKDL occur after infection with *Leishmania donovani*, less commonly so after *L. infantum *and extremely rarely following infection by**Leishmania* chagasi *(Singh *et al.*, 2011). PKDL in HIV co-infected patients is more common and more severe, and is not restricted to *L. donovani *(Zijlstra, 2014). After the first reports from the coasts of the Mediterranean basin in the 1990s, HIV and *Leishmania* co-infection has been increasingly reported (Monge-Maillo *et al.*, 2014). In Serbia, this co-infection still appears to be rare and includes one documented case of VL/HIV co-infection in Niš, south Serbia, caused by *L. infantum* as confirmed by molecular analyses of the parasites identified from blood (Marjanović *et al.*, 2012), and two cases identified in our clinic.

Introduction of molecular diagnostics for patients with VL in Serbia is warranted. Molecular diagnosis of VL using DNA extracted from unstained BMSs stored for several years is possible. This is especially useful in the diagnosis of complicated VL cases, for which unstained BMSs could easily be mailed to centres where PCR is available. It is also useful for retrospective epidemiological studies.

## References

[R1] Beatović V.(2010). The Institute of Public Health of Montenegro, The Ministry ofHealth of Montenegro, *WHO Exploratory Meeting on Leishmaniasis in the Balkan Countries*, Dubrovnik, Croatia, 10–12 February 2010. Available on: (http://www.who.int/leishmaniasis/resources/MONTENEGRO.pdf).

[R2] Brustoloni Y. M. Lima R. B. Da Cunha R. V. Dorval M. E. Oshiro E. T. De Oliveira A. L. Pirmez C.(2007). Sensitivity and specificity of polymerase chain reaction in Giemsa-stained slides for diagnosis of visceral leishmaniasis in children. Mem Inst Oswaldo Cruz 102 497–500. 10.1590/S0074-02762007005000036 17612771

[R27] Cirović D. Chochlakis D. Tomanović S. Sukara R. Penezić A. Tselentis Y. Psaroulaki A.(2014). Presence of *Leishmania *and *Brucella *species in the golden jackal *Canis aureus* in Serbia. Biomed Res Int 2014 728516. 10.1155/2014/728516 24967397 PMC4055068

[R3] Cortes S. Rolão N. Ramada J. Campino L.(2004). PCR as a rapid and sensitive tool in the diagnosis of human and canine leishmaniasis using *Leishmania donovani s.l.*-specific kinetoplastid primers. Trans R Soc Trop Med Hyg 98 12–17. 10.1016/S0035-9203(03)00002-6 14702834

[R25] Da Silva E. S. Gontijo C. M. Pacheco R. S. Brazil R. P.(2004). Diagnosis of human visceral leishmaniasis by PCR using blood samples spotted on filter paper. Genet Mol Res 3 251–257. 15266395

[R4] Dakic Z. D. Pelemis M. R. Stevanovic G. D. Poluga J. L. Lavadinovic L. S. Milosevic I. S. Indjic N. K. Ofori-Belic I. V. Pavlovic M. D.(2009). Epidemiology and diagnostics of visceral leishmaniasis in Serbia. Clin Microbiol Infect 15 1173–1176. 10.1111/j.1469-0691.2009.02768.x 19392902

[R5] Das N. K. Singh S. K. Ghosh S. Sarkar A. Mukhopadhyay D. Roy S. Ganguly D. N. Barbhuiya J. N. Saha B. Chatterjee M.(2011). Case series of misdiagnosis with rK39 strip test in Indian leishmaniasis. Am J Trop Med Hyg 84 688–691. 10.4269/ajtmh.2011.10-0590 21540376 PMC3083734

[R26] De Ruiter C. M. Van der Veer C. Leeflang M. M. Deborggraeve S. Lucas C. Adams E. R.(2014). Molecular tools for diagnosis of visceral leishmaniasis: systematic review and meta-analysis of diagnostic test accuracy. J Clin Microbiol 52 3147–3155. 10.1128/JCM.00372-14 24829226 PMC4313130

[R6] Durda A.(2010). WHO Exploratory Meeting on Leishmaniasis in the Balkan Countries. Dubrovnik, Croatia, 10–12 February 2010 http://www.who.int/leishmaniasis/resources/BOSNIA.pdf.

[R7] Ganguly S. Das N. K. Barbhuiya J. N. Chatterjee M.(2010). Post-kala-azar dermal leishmaniasis - an overview. Int J Dermatol 49 921–931. 10.1111/j.1365-4632.2010.04558.x 21128917

[R8] Gebhardt M. Ertas B. Falk T. M. Blödorn-Schlicht N. Metze D. Böer-Auer A.(2015). Fast, sensitive and specific diagnosis of infections with *Leishmania* spp. in formalin-fixed, paraffin-embedded skin biopsies by cytochrome b polymerase chain reaction. Br J Dermatol 173 1239–1249. 10.1111/bjd.14088 26286104

[R9] Gradoni L.(2013). Epidemiological surveillance of leishmaniasis in the European Union: operational and research challenges. Euro Surveill 18 20539. 10.2807/1560-7917.ES2013.18.30.20539 23929176

[R10] Haldar J. P. Saha K. C. Ghose A. C.(1981). Serological profiles in Indian post kala-azar dermal leishmaniasis. Trans R Soc Trop Med Hyg 75 514–517. 10.1016/0035-9203(81)90188-7 7324125

[R11] Institute of Public Health of Serbia(2009). *Report on Infectious Diseases in the Republic of Serbia in 2008*, p. 28 (in Serbian). Available on: (http://www.batut.org.rs/index.php?content=299).

[R12] Institute of Public Health of Serbia(2015). *Report on Infectious Diseases in the Republic of Serbia in 2014*, p. 63 (in Serbian). Available on: (http://www.batut.org.rs/download/izvestaji/Izvestaj%20o%20zaraznim%20bolestima%202014.pdf).

[R13] Ivović V. Depaquit J. Léger N. Urano A. Papadopoulos B.(2004). Sandflies (Diptera: Psychodidae) in the bar area of Montenegro (Yugoslavia). 2. Presence of promastigotes in *Phlebotomus neglectus* and first record of *P. kandelakii*. Ann Trop Med Parasitol 98 425–427. 10.1179/000349804225003352 15228724

[R14] Lanús E. C. Piñero J. E. González A. C. Valladares B. De Grosso M. L. Salomón O. D.(2005). Detection of *Leishmania braziliensis* in human paraffin-embedded tissues from Tucumán, Argentina by polymerase chain reaction. Mem Inst Oswaldo Cruz 100 187–192. 10.1590/S0074-02762005000200013 16021307

[R15] Lipoldová M. Demant P.(2006). Genetic susceptibility to infectious disease: lessons from mouse models of leishmaniasis. Nat Rev Genet 7 294–305. 10.1038/nrg1832 16543933

[R16] Mahmoudzadeh-Niknam H. Abrishami F. Doroudian M. Moradi M. Alimohammadian M. Parvizi P. Hatam G. Mohebali M. Khalaj V.(2011). The problem of mixing up of *Leishmania *isolates in the laboratory: suggestion of ITS1 gene sequencing for verification of species. Iran J Parasitol 6 41–48. 22347273 PMC3279869

[R17] Marjanovic G. Miladinovic-Tasic N. Gabrielli S. Otasevic S. Popovic-Dragonjic L. Kocic B. Arsic-Arsenijevic V. Tadic L. Cancrini G.(2012). First case of visceral leishmaniosis/HIV coinfection in Nis - southeastern Serbia. Archives Biological Sci 64 1271–1276. 10.2298/ABS1204271M

[R18] Monge-Maillo B. Norman F. F. Cruz I. Alvar J. López-Vélez R.(2014). Visceral leishmaniasis and HIV coinfection in the Mediterranean region. *PLoS Negl Trop *Dis 21 e3021 10.1371/journal.pntd.0003021 PMC4140663 25144380

[R19] Peacock C. S. Seeger K. Harris D. Murphy L. Ruiz J. C. Quail M. A. Peters N. Adlem E. Tivey A.(2007). Comparative genomic analysis of three *Leishmania* species that cause diverse human disease. Nat Genet 39 839–847. 10.1038/ng2053 17572675 PMC2592530

[R20] Savić S. Vidić B. Grgić Z. Potkonjak A. Spasojevic L.(2014). Emerging vector-borne diseases - incidence through vectors. Front Public Health 2 267. 10.3389/fpubh.2014.00267 25520951 PMC4251170

[R21] Singh S. Sharma U. Mishra J.(2011). Post-kala-azar dermal leishmaniasis: recent developments. Int J Dermatol 50 1099–1108. 10.1111/j.1365-4632.2011.04925.x 22126871

[R28] Šiško-Kraljević K. Jerončić A. Mohar B. Punda-Polić V.(2013). Asymptomatic *Leishmania infantum* infections in humans living in endemic and non-endemic areas of Croatia, 2007 to 2009. Euro Surveill 18 20533. 10.2807/1560-7917.ES2013.18.29.20533 23929119

[R22] Vaselek S. Savić S. Di Muccio T. Erisoz Kasap O. Gradoni L. Alten B. Petrić D.(2015). Possible re-emergence of leishmaniasis in Serbia. In *Abstracts of the 2nd Conference on Neglected Vectors and Vector-Borne Diseases with MC and WG Meetings of the COST Action TD1303*, Izmir, Turkey, 31 March–2 April 2015, p. 4 (http://eurnegvec.org/2ac_abstractbook.pdf). 10.1186/s13071-017-2386-z PMC5615930 28950895

[R24] Zijlstra E. E.(2014). PKDL and other dermal lesions in HIV coinfected patients with leishmaniasis: review of clinical presentation in relation to immune responses. PLoS Negl Trop Dis 20, e3258. 10.1371/journal.pntd.0003258 PMC4238984 25412435

[R23] Zijlstra E. E. Musa A. M. Khalil E. A. El-Hassan I. M. El-Hassan A. M.(2003). Post-kala-azar dermal leishmaniasis. Lancet Infect Dis 3 87–98. 10.1016/S1473-3099(03)00517-6 12560194

